# PP31 Logical Model Articulating Pharmacy Commissions And Hospital-Based Health Technology Assessment Nuclei: Collaborative Strategies For The Drug Selection

**DOI:** 10.1017/S0266462324002046

**Published:** 2025-01-07

**Authors:** Denise Souza Stopatto, Flávia Tavares Silva Elias

## Abstract

**Introduction:**

Collaborative strategies between the Pharmacy and Therapeutics Committee (Group 1) and hospital-based health technology assessment (HTA) nuclei (Group 2) using HTA tools can promote rational use and medicines access. The aim was to develop a logical model for the drug selection process in Brazilian university hospitals, considering the articulation between the Pharmacy and Therapeutics Committee and hospital-based HTA nuclei (NATS).

**Methods:**

A qualitative study created and validated a logical model. The data collection considered documental analysis and semistructured interviews with key informants from both groups about the processes adopted by eight federal university hospitals. The European Project on Hospital-Based Health Technology Assessment (AdHopHTA) was selected as a theoretical reference. The model was discussed in a focal group with the participation of five experts in medicine selection tasks and HTA. The final version of the model was submitted for consistency and vulnerability analysis by the authors. The ethical approval number is 4.784.861.

**Results:**

The logical model was conceived with dimensions of resources, actions, products, results, and impacts. It has allowed a structured and organized view of the 15 actions in the medicine selection. From these 15 actions, seven can be articulated between both groups. The high management support, training, and dissemination of the procedures and flows that organize HTA processes were solutions to minimizing collaborative barriers. The selection of medicines was considered a dynamic process. Some actions, such as the use of pharmacovigilance data, post-selection monitoring, and horizon technology scanning can bring results in terms of treatment innovations and compliance with clinical guidelines.

**Conclusions:**

Collaborative strategies were presented in a logical model with seven joint actions to guide the medicine selection process in hospitals involving tools, flow audits, governance, and human resources. The consistency and vulnerability model analysis defined indicators for HTA collaborative actions.

